# Establishment of a mouse xenograft model of metastatic adrenocortical carcinoma

**DOI:** 10.18632/oncotarget.16909

**Published:** 2017-04-07

**Authors:** Aurélie Morin, Carmen Ruggiero, Estelle Robidel, Mabrouka Doghman-Bouguerra, Atze T. Das, Rémy Castellano, Emmanuelle Josselin, Judith Favier, Enzo Lalli

**Affiliations:** ^1^ Université Paris Descartes, Sorbonne Paris Cité, Paris, France; ^2^ Inserm UMR970, Paris Cardiovascular Research Centre, Paris, France; ^3^ Université Côte d’Azur, Valbonne, Sophia Antipolis, France; ^4^ Institut de Pharmacologie Moléculaire et Cellulaire, Valbonne, Sophia Antipolis, France; ^5^ Laboratory of Experimental Virology, Department of Medical Microbiology, Academic Medical Center, University of Amsterdam, Amsterdam, The Netherlands; ^6^ Aix Marseille University, CNRS, INSERM, Institut Paoli-Calmettes, CRCM, Marseille, France

**Keywords:** adrenal cortex, cancer, cell lines, mouse models, xenografts

## Abstract

Adrenocortical carcinoma is a rare neoplasm with a poor prognosis. Very important advances have been made in the identification of the genetic determinants of adrenocortical carcinoma pathogenesis but our understanding is still limited about the mechanisms that determine cancer spread and metastasis. One major problem hindering preclinical experimentation for new therapies for adrenocortical carcinoma is represented by the lack of suitable animal models for metastatic disease. With the aim to overcome these limitations, in this study we tested several protocols in order to establish a mouse xenograft model of metastatic adrenocortical carcinoma. The most efficient method, based upon intrasplenic injection followed by splenectomy, produced metastases with high efficiency, whose development could be followed over time by bioluminescence measurements. We expect that the availability of this model will greatly improve the possibilities for preclinical testing of new treatments for advanced-stage disease.

## INTRODUCTION

Malignant adrenal tumours represent a severe medical condition. Among them, adrenocortical carcinoma (ACC) is a rare neoplasm with a poor prognosis since it is often diagnosed already at the metastatic stage. Decisive advances have been recently made in the identification of the genetic determinants of ACC pathogenesis [1–3] but the mechanisms driving its spread and metastasis remain largely unknown. Metastatic dissemination is a complex, dynamic and inefficient process involving multiple steps, which is responsible for more than 90% of fatal events in cancer patients [4]. Liver and lungs are the most common sites of ACC metastases. A cornerstone of the therapy for metastatic ACC is the use of mitotane, a derivative of the insecticide DDT [5], which can be associated to cytotoxic chemotherapy. However, current therapies for ACC are unable to significantly increase overall survival of patients [6]. A consensus exists that new, more active pharmacological tools are urgently needed for the treatment of metastatic ACC. Furthermore, modern therapies for those tumours should be tailored to the specific biological features of each cancer, as it is currently a standard for other types of cancer with the goal of a personalized therapy.

One major problem hindering preclinical experimentation of new therapies for ACC is represented by the lack of suitable animal models for metastatic disease. Current protocols envisage the use of human cell lines or patient-derived tumour fragments as xenografts implanted in immunodeficient mice [7, 8]. However, those xenografts have no metastatic potential and tests of new pharmacological therapies are restricted to targeting of the primary tumour [9–19]. Genetic models of ACC also exist but they are limited by their poor tendency to diffuse beyond the loco-regional stage [20, 21].

To overcome these limitations, in this study we tested several grafting procedures in order to establish a model of metastatic ACC.

## RESULTS

### Generation of the H295R/TR SF-1 GFP-luc cell line

**Figure 1:**
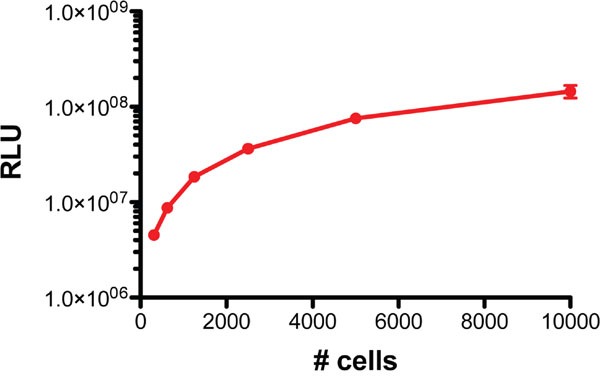
Luminescence of H295R/TR SF-1 GFP-luc cells Various numbers of cells (1:2 dilutions starting from 10^4^ to 312/well) were seeded in triplicate in a 96-well plate and luminescence measured by an *in vitro* luciferase assay.

Human ACC H295R/TR SF-1 cells [22] were transduced with a lentiviral vector and selected by cell sorting to generate a cell line expressing both firefly luciferase and GFP for bioluminescence and fluorescence detection. Luciferase activity from this cell line (H295R/TR SF-1 GFP-luc) is easily detectable *in vitro* from a low number of cells (Figure 1).

### Caudal vein injection

In the attempt to develop a metastatic mouse model of ACC, we first explored the potential of intravenous injection of cancer cells into the caudal vein [23]. However, out of ten mice receiving H295R/TR SF-1 GFP-luc cells using this procedure, only one displayed small foci detectable by bioluminescence at the level of kidneys and lung 43 days after the injection (Supplementary Figure 1).

### Renal subcapsular grafts

**Figure 2:**
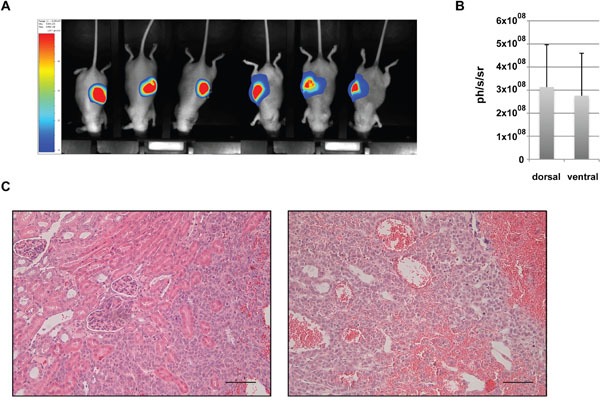
Analysis of renal subcapsular xenografts **(A)** Representative bioluminescence images and **(B)** quantitative analysis of photon counts derived from renal subcapsular xenografts of H295R/TR SF-1 GFP-luc cells. **(C)** Representative images of HES-stained sections of renal subcapsular tumours at 52 days after cell graft. Note the large vascular spaces inside the xenograft tissue. Scale bars, 50 μm.

Thirteen mice were grafted under the renal capsule using 2.7×10^6^ H295R/TR SF-1 GFP-luc cells per animal. Bioluminescence imaging to detect primary tumour and potential metastases was performed 43 days after surgery just before the sacrifice of the animals. All mice showed a strong bioluminescent signal near the left kidney, that was comparable in intensity whether the mice were exposed on their ventral or dorsal face (Figure 2A, 2B). The intensity of the bioluminescent signal did not correlate with the weight of the tumor mass (r^2^ = 0.15), probably due to the high heterogeneity of the tumours revealed by histological analysis (Figure 2C). Imaging analysis did not reveal any potential metastatic site and no macroscopic metastasis could be observed at necropsy.

### Intrasplenic grafts

**Figure 3:**
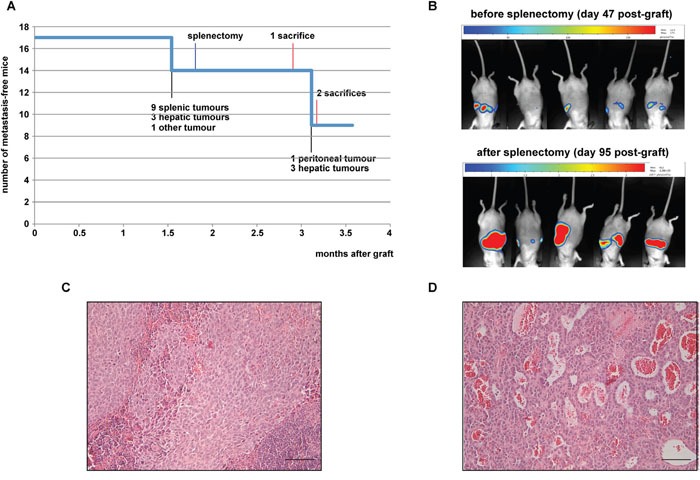
Analysis of intrasplenic xenografts **(A)** Number of mice without metastases detectable by bioluminescence imaging in function of time after graft. Milestones of the experiment are indicated. **(B)** Representative bioluminescence images of mice 47 and 94 days after intrasplenic xenograft of H295R/TR SF-1 GFP-luc cells (before and after splenectomy, respectively). **(C)** A representative HES-stained section of a splenic tumour at 54 days after cell graft. **(D)** A representative HES-stained section of a hepatic metastasis 109 days after cell graft. Note the large vascular spaces inside the metastasis. Scale bars, 50 μm.

Because of the lack of success of the previous protocols, we chose to inject H295R/TR SF-1 GFP-luc cells intrasplenically, to elicit formation of hepatic metastases. We injected 5×10^6^ H295R cells into the spleen of 17 mice and monitored tumour burden by bioluminescence imaging. 47 days after the graft, 9 mice presented a bioluminescence signal indicative of a splenic tumour. Among those mice, 3 also had a detectable signal in the upper part of the abdomen, suggestive of a hepatic tumour, and one had a signal in the lower part of the abdomen (Figure 3A, 3B). To allow metastasis progression before sacrifice, mice underwent radical splenectomy 54 days after the graft. Only 6 mice (out of the 9 mice having bioluminescent signal) had a macroscopically evident splenic tumour, highlighting the sensitivity of bioluminescence imaging over macroscopic observation. Histological analysis of the splenic tumours showed nearly complete replacement of lymphoid tissue by xenograft (Figure 3C). Tumour burden was monitored by bioluminescence imaging and mice were sacrificed either when the identified hepatic tumour reached the luminescence of 6×10^8^ ph/s/sr or 109 days after the graft. At the end of the experiment, 6 mice out of 17 had liver metastasis detected by imaging and confirmed by histology (Figure 3D), one had a pancreatic tumour and a tumour linked to the peritoneal muscle (both tumours very close to the injection site). Another mouse also presented a peritoneal tumour next to the injection site. Both primary tumours and liver metastases expressed the adrenocortical marker SF-1 and displayed a high Ki67 labelling index (data not shown).

### Intrasplenic grafts followed by splenectomy

**Figure 4:**
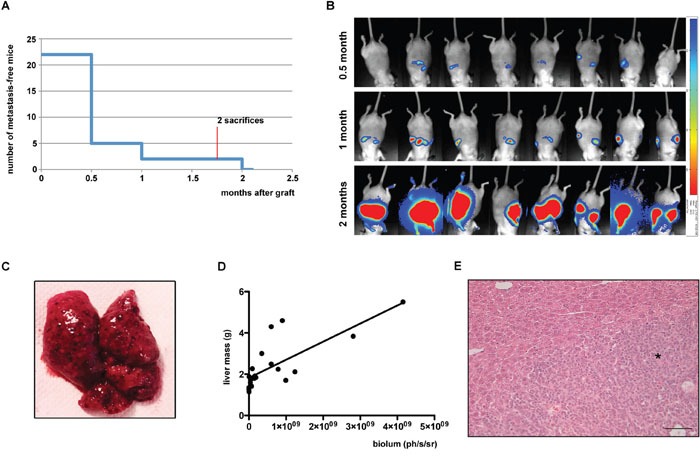
Analysis of intrasplenic xenografts complemented by splenectomy **(A)** Number of mice without metastases detectable by bioluminescence imaging in function of time after graft. **(B)** Representative *in vivo* images from bioluminescence imaging of mice 0.5, 1 or 2 months after intrasplenic xenograft of H295R/TR SF-1 GFP-luc cells followed by splenectomy. **(C)** Macroscopic image of a liver from a mouse injected with H295R/TR SF-1 GFP-luc cells in the spleen, showing multiple metastatic nodules. **(D)** Correlation between liver mass at necropsy and intensity of bioluminescence signals measured 2 months after graft. r^2^= 0.5793, p<0.0001. **(E)** HES-stained section of a hepatic metastasis 2 months after cell graft. Metastatic tissue is indicated with an asterisk. Scale bar, 50 μm.

To increase tumour burden we then decided to increase the number of grafted cells. However, to avoid the development of tumours around the injection site (pancreas or peritoneum), we decided to splenectomize all mice during the initial surgery. We grafted 22 mice with 10^7^ H295R/TR SF-1 GFP-luc cells through the intrasplenic route and subsequently we ligatured and resected the spleen 10 minutes after injection. At 1 month after cell graft, 20 mice (91%) presented tumours in the upper part of the abdomen detectable by bioluminescence imaging. At the end of the experiment 2 months after graft, all mice had bioluminescence detectable and macroscopically visible hepatic tumours (Figure 4A-4C). A significant linear correlation existed between liver weight at necropsy and the intensity of bioluminescent signals (Figure 4D). No other kind of tumour was observed at necropsy. Histological analysis of these tumours revealed strong metastatic invasion of hepatic tissue (Figure 4E). Metastatic nodules retained their adrenocortical differentiation, as shown by SF-1 staining, and displayed a high Ki67 labelling index, in contrast to the surrounding liver tissue (Supplementary Figure 2).

## DISCUSSION

Until very recently, H295R cells and its derivatives were the only differentiated human ACC cell line available for *in vitro* and xenograft studies. New perspectives to better appreciate ACC heterogeneity may now be offered by the recently described SJ-ACC3 xenografts [24] and MUC-1 xenografts and cell line [19]. Nevertheless, H295R cells remain the workhorse among human ACC models. While retaining steroidogenic capacities, these cells have multiple features of a highly malignant phenotype, including among others a very complex karyotype (our unpublished observations), *TP53* [22] and *CTNNB1* [25] mutations. However, these characteristics do not translate into the potential of this cell line to spontaneously metastasize when grown as xenografts in the highly vascularized environment under the renal capsule. The metastatic process requires multiple steps, from the capacity of the tumour cells to reach the blood and lymphatic circulation to the ability to extravasate and colonize peripheral organs [26]. H295R cells are clearly defective in one or more of these essential steps for tumour spread and metastasis, which will be worthwhile to precisely identify in future studies. We acknowledge that intrasplenic graft is an experimental model of metastasis and not a spontaneous one. As such, it eludes the stages of local invasion and intravasation. However, intrasplenic graft is a validated experimental model of liver metastasis, representative of the later stages of the malignant process: circulation to a distant organ, arrest and extravasation, followed by establishment of micrometastasis and metastatic colonization. Since the two last steps are often considered as rate-limiting in the malignant process [27, 28], the establishment of a model recapitulating those steps appears valuable for the understanding of human disease.

In many types of epithelial cancer, cells transition to a mesenchymal-like phenotype (EMT) is of high relevance for invasion and metastasis [29]. It has been reported that loss of β-catenin induces reversal of EMT in H295R cells, as shown by downregulation of vimentin, N-cadherin and Slug upon *CTNNB1* knockdown [30]. However, mesoderm-derived tissues such as the adrenal cortex normally express vimentin, while N-cadherin was reported to be downregulated in ACC compared to adrenocortical adenomas [31]. It thus remains uncertain whether a process related to EMT really exists and has relevance for metastatic dissemination in ACC.

In this study, by comparison of different procedures, we have developed a protocol to induce liver metastasis in immunodeficient mice grafted with the human H295R/TR SF-1 GFP-luc ACC cell line. This method, based upon intrasplenic injection followed by splenectomy, produced metastases in the liver with 100% efficiency, whose development could be followed over time by bioluminescence measurements. Early splenectomy reduces the manipulation of animals, eliminating the need for a second surgery, and avoids the development of primary splenic tumours, which allows time for liver metastases to develop. The number of engrafted cells may be an important factor for the success of intrasplenic injection compared to the other tested routes for tumour cells administration, eliminating the number of cells or injection volume constraints imposed by caudal vein injection and subcapsular renal graft, respectively On the other hand, one limitation of our model is that no metastases were observed in the other organs mainly targeted by ACC metastasis, i.e. lungs and bone. However, caudal vein injection of tumor cells was not efficient in producing lung metastasis and experimental metastases to the bone are usually obtained by intracardiac injection, a technically demanding and high-risk procedure. Conversely, the method described here can be easily performed with no loss of animals caused by complications of surgery. We expect that the availability of this mouse xenograft model of metastatic ACC will greatly improve the possibilities for preclinical testing of new treatments for advanced-stage disease.

## MATERIALS AND METHODS

### Cell culture

H295R/TR SF-1 cells, derived from the H295R cell line [32], were generated in our laboratory [22] and reauthenticated by STR profiling (Promega PowerPlex 21 PCR Kit) immediately prior to use for this work. Cell were cultured in a humidified atmosphere containing 5% CO_2_ at 37°C in DMEM/F12 complemented with 2% NuSerum (BD Biosciences), 1% ITS+ (BD Biosciences), blasticidin (5 μg/ml, Cayla InvivoGen) zeocin (100 μg/ml, Cayla InvivoGen), and penicillin/streptomycin (Invitrogen), as described [22].

### Lentiviral infection and production of H295R/TR SF-1 GFP-luc cells

The pRRL-GFP/Luc2 vector [33] was packaged into lentiviral particles and used to infect H295R/TR SF-1 cells in the exponential phase of growth seeded in a 6-well plate at a density of 1×10^6^ cells/well. The following day, culture medium was replaced by fresh medium containing polybrene (Sigma Aldrich #H9268) at a final concentration of 4 μg/ml. After 1 hour, cells were incubated for 24 hours with complete culture medium (1.5 ml/well) containing 5×10^6^ virions and 4 μg/ml polybrene. The following day the medium was replaced and the efficiency of infection was determined by monitoring the expression level of GFP under an inverted fluorescent microscope. Cells were amplified by sequential passage and then purified by FACS. Before cell sorting, cells were filtered through a 70 μM strainer to eliminate all remaining clumps and resuspended at a concentration of 8×10^6^/ml. About 24 millions cells were sorted using a FACSARIA III instrument (BD Biosciences). A highly pure sorting mode (4-way purity sorting) was chosen. Sorted cells expressing the highest levels of GFP fluorescence (around 4% of the initial population) were collected in polypropylene tubes containing FBS, plated in 25 cm^2^ cell culture flasks and amplified. GFP expression in the polyclonal population obtained was confirmed by fluorescence microscopy. To measure luciferase expression, various numbers of cells were seeded in the wells of white 96-well plates (Corning) in triplicate in 100 μl of culture medium per well. After cell lysis, luciferase activity was measured using the Luciferase Assay System (Promega #E1500) on a GloMax instrument (Promega).

### Animal experiments

All mouse husbandry and experimental procedures were performed in accordance with protocols 2–091009 and 14-041 approved by the local Committees for Animal Experimentation and followed the ARRIVE guidelines of the National Centre for the Replacement, Refinement, and Reduction of Animals in Research (London, UK). They were carried out using female MRI Nude mice (Janvier) aged 6 to 16 weeks. Animals had free access to water and standard laboratory chow, and were housed according to institutional rules with 12:12 h dark/light cycles. For procedures involving surgery, mice were anesthetized by inhalation of isoflurane and were given meloxicam (2 mg/kg s.c.) analgesia after the surgical intervention. For 1 week after surgery, animals were given a broad-spectrum antibiotic (enrofloxacin 100 μg/ml) in drinking water supplemented with sucrose.

### Caudal vein injection

1×10^6^ H295R/TR SF-1 GFP-luc cells resuspended in 100 μl of a 0.9% NaCl solution were injected into the mouse caudal vein using a 29G needle.

### Renal subcapsular graft

H295R/TR SF-1 GFP-luc cells were trypsinized, centrifuged and gently resuspended in 30 μl ice-cold PBS per injection. An equal volume of ice-cold Matrigel (high concentration, catalog number 354248, BD Biosciences) was added. Mice were anesthetized and the left kidney was exposed by a retroperitoneal incision and gently lifted to allow the injection of the cells under the renal capsule. The kidney was kept humid during the procedure to avoid renal capsule rupture. 2.7×10^6^ cells were injected using a 29G needle. The needle was slowly inserted under the renal capsule with the bevelled edge facing up. Air was first injected to separate the capsule from the kidney, and consequently 40 μl of cell suspension was injected. The needle was left immobile for 1 minute to allow Matrigel polymerization, then turned to face down the bevel, and carefully withdrawn from the kidney to prevent reflux of the injected cells.

### Intra-splenic injection

5 -10×10^6^ H295R cells were resuspended in 60 μL PBS and injected at the inferior pole of the spleen using a 29G needle. The bevel of the needle was observed through the splenic capsule to avoid injecting the cells under the spleen. Whitening of the spleen and blood vessels was observed upon injection. When stated, mice underwent splenectomy through ligature of the pancreas and splenic vessels and complete resection of the spleen.

### Bioluminescence imaging

At various times after the beginning of the different protocols, after isoflurane anaesthesia mice were injected intraperitoneally with 150 mg/kg body weight of endotoxin-free beetle luciferin, potassium salt (Promega) solution in PBS 8 minutes prior to imaging. Bioluminescence measurements were performed using a PhotonIMAGER instrument (Biospace Lab). Data were acquired for 11 minutes and analyzed using the M3 vision software (Biospace Lab).

### Immunohistochemistry

Immunohistochemistry was performed as described [34], using the anti SF-1 (clone N1665, Perseus Proteomics) and anti Ki67 (clone SP6, Thermo Fisher) antibodies.

### Statistical analysis

Linear regression was calculated using the GraphPad Prism software v. 5.0d.

## SUPPLEMENTARY MATERIALS FIGURES



## Abbreviations

ACC: adrenocortical carcinoma
EMT: epithelial-mesenchymal transition
GFP: green fluorescent protein
HES: hematoxylin-eosin-safran
SF-1: Steroidogenic Factor 1
