# Supplementary material for: Establishment of a mouse xenograft model of metastatic adrenocortical carcinoma
Source: Oncotarget. 2017 Apr 7;8(31):51050–7. doi: 10.18632/oncotarget.16909 (PMC5584229; doi:10.18632/oncotarget.16909)
Supplement: Supplementary file 1 [file oncotarget-08-51050-s001.pdf]

Establishment of a mouse xenograft model of metastatic adrenocortical carcinoma

SUPPLEMENTARY MATERIALS

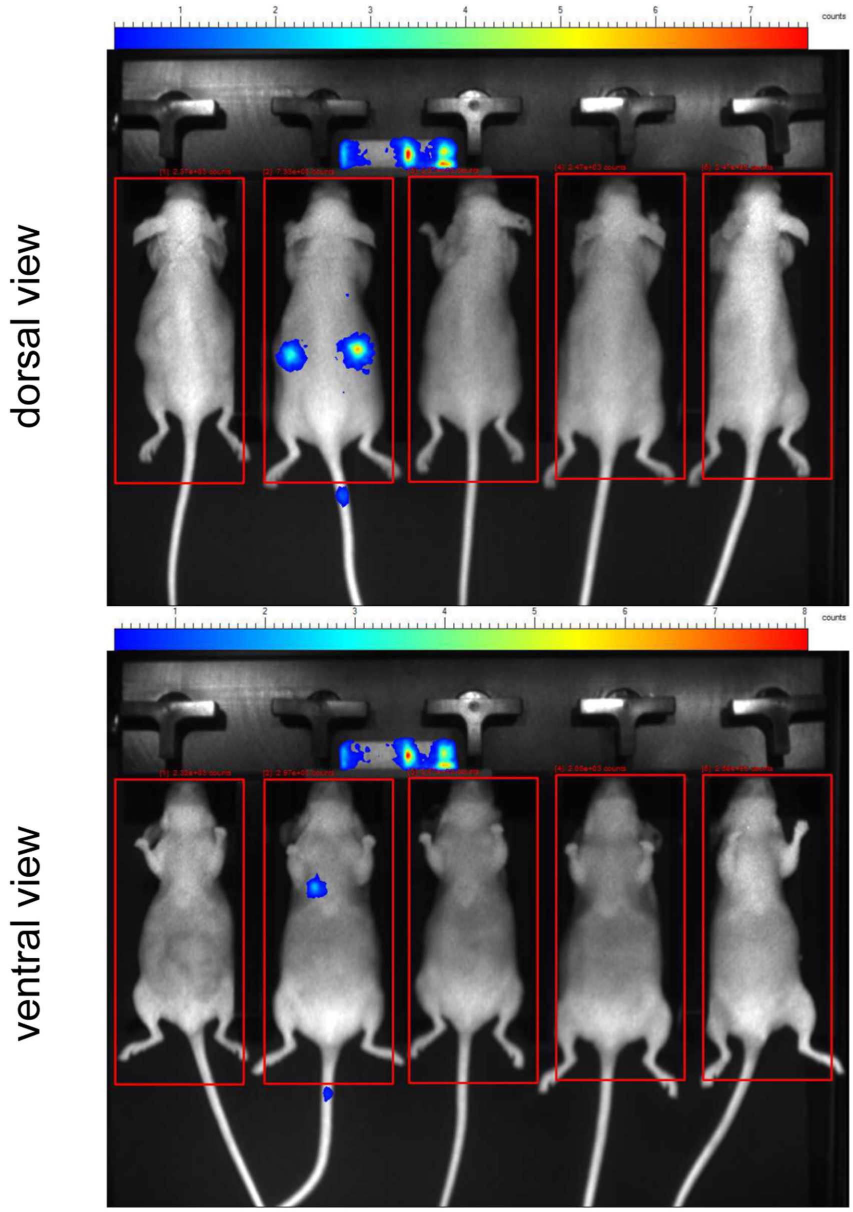

Supplementary Figure 1: Bioluminescence of immunodeficient mice that received H295R/TR SF-1 GFP-luc cells injection in the caudal vein. Images were taken 43 days after the injection. Dorsal and ventral views.

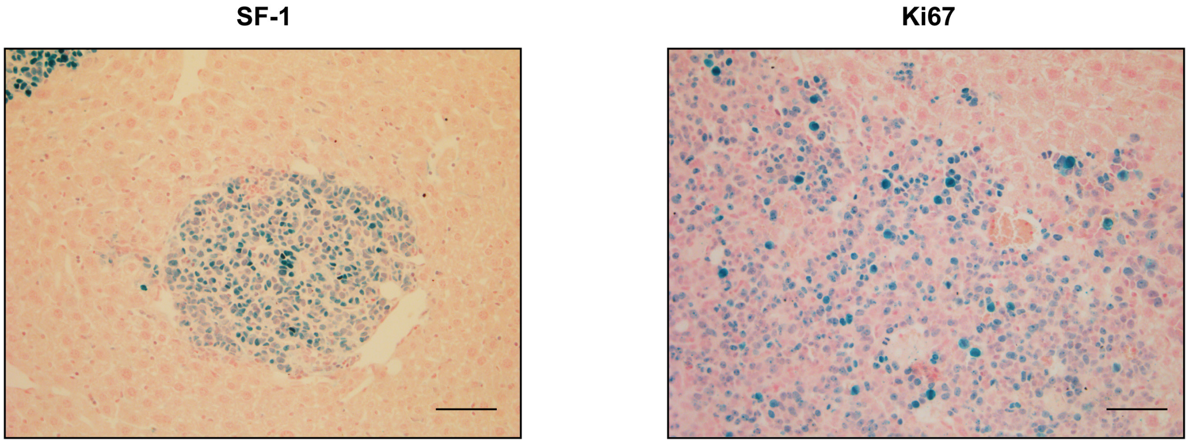

Supplementary Figure 2: Metastatic nodules in the liver express the adrenocortical marker SF-1 and have a high KI67 labeling index. Scale bars, 50 µm.
